# Neurocognitive Profiles of Caucasian Moyamoya Disease Patients in Greece: A Case Series

**DOI:** 10.3390/neurosci3010010

**Published:** 2022-02-23

**Authors:** Georgios Papageorgiou, Dimitrios Kasselimis, Georgia Angelopoulou, Dimitrios Tsolakopoulos, Nikolaos Laskaris, Argyro Tountopoulou, Eleni Korompoki, Georgios Velonakis, Achilles Chatziioannou, Konstantinos Spengos, Constantin Potagas, Sophia Vassilopoulou

**Affiliations:** 1 Neuropsychology and Language Disorders Unit, 1st Department of Neurology, National and Kapodistrian University of Athens, Eginition Hospital, 15772 Athens, Greece; dkasselimis@gmail.com (D.K.); georginangel@gmail.com (G.A.); dimitris_tsolakopoulos@hotmail.com (D.T.); cpotagas@otenet.gr (C.P.); 2 Department of Industrial Design and Production Engineering, School of Engineering, University of West Attica, 12243 Athens, Greece; nikolaos.laskaris@gmail.com; 3 Stroke Unit, 1st Department of Neurology, National and Kapodistrian University of Athens, Eginition Hospital, 15772 Athens, Greece; atounto@yahoo.gr (A.T.); e.korompoki@imperial.ac.uk (E.K.); svassilopoulou@gmail.com (S.V.); 4 Department of Clinical Therapeutics, National and Kapodistrian University of Athens, 15772 Athens, Greece; 5 2nd Department of Radiology, National and Kapodistrian University of Athens, 15772 Athens, Greece; giorvelonakis@gmail.com; 6 1st Department of Radiology, National and Kapodistrian University of Athens, 15772 Athens, Greece; achatzi@med.uoa.gr; 7 Department of Neurology, Hygeia Hospital, 15123 Athens, Greece; kspengos@hygeia.gr

**Keywords:** moyamoya, stroke, executive functions, working memory, social cognition

## Abstract

The impact of Moyamoya Disease (MMD) on cognition inadult Caucasian patients has not yet been thoroughly investigated. The current study is the first to present detailed neuropsychological data on a series of Greek patients with MMD. A group of eight patients was assessed with an extensive neuropsychological battery, including measures of episodic memory, working memory, executive functions, language, and social cognition. The results indicated that MMD may be characterized by a trichotomous neurocognitive profile, characterized by prominent impairment in working memory, executive functions, and social cognition. Overall, we stress the need for a thorough cognitive evaluation of MMD patients and further highlight the potential importance of social cognition in this particular disease. Possible associations between the three impaired cognitive domains in our group are also discussed.

## 1. Introduction

Moyamoya disease (MMD) is a degenerative, progressive cerebrovascular disorder characterized by blockage of the internal carotid artery at the Circle of Willis, resulting in the formation of collaterals that have a distinct cloudy appearance on angiography [1]. However, the etiology and pathogenesis of MMD still remains unclear. In recent years, there is growing evidence indicating circulating and genetic factors related to MMD’s onset and course. As for the circulating factors, they mainly involve endothelial progenitor cells, polymorphisms of cytokines, and the dysfunction of caveolin [2]. Genetic predisposition also seems to be of importance for the occurrence of MMD, while racial differences are observed as well. Several studies in Japanese families with familial MMD exhibit linkage to at least five chromosomal regions: 3p24.2-p26,26 6q25,27 8q23,59 12p12,59 and 17q25.46,79. More specifically, the *RNF213* gene p.R4810K variant was characterized as the susceptibility gene in the East Asian population, but different variants of the same gene in non-p.R4810K were identified in the United States and Caucasian cases [3].As for the cognitive profiles of patients with MMD, neuropsychological implications have been described in children [4]. Nevertheless, few studies have examined neurocognitive functioning in adults with MMD. In addition, the incidence of MMD is extremely rare in Caucasians [5], resulting in even fewer studies on neurocognitive findings in European patients. Existing studies tend to limit their cognitive testing to measures of intelligence, providing only general information about cognition in MMD. Thus, the existing literature lacks any specifics on the effect of the disease on distinct cognitive domains. Published papers with adult patients are mostly case studies [6,7,8] reporting neuropsychological findings in presurgical single patients. Such cases have been found to exhibit low intelligence scores [9], visuospatial ability [7], as well as executive functions such as processing speed [6,7,10], while memory functioning is usually found to be intact [8]. Scarce studies with larger groups of patients seem to confirm the aforementioned pattern shown in single case reports by highlighting prominent deficits in executive functions, but also in language, while memory appears to be generally preserved [11]. It should, however, be noted that stroke may serve as a confounding factor on the neuropsychological profile, as it could selectively affect specific cognitive domains, depending on lesion topology. Given that MMD patients without stroke have been shown to exhibit cognitive impairment similar to that of MMD patients with stroke [12], the implications of cerebrovascular accident(s) in this particular clinical population have not yet been fully elucidated.

Another issue related to the above-described scarcity of detailed neuropsychological data in MMD is that most published reports focus on a limited number of cognitive domains and seem to ignore important aspects of behavior such as measures of social cognition. We argue that such an exclusion poses a limitation to the detailed description of MMD patients’ cognitive profile based on previous studies’ hypotheses in favor of the relationship between executive functions and aspects of social cognition, such as theory of mind. It should be also noted that there are indications of theory of mind deficits in the clinical descriptions of such patients in published reports, as well as anecdotal evidence from clinical practice suggesting that at least some of these patients may exhibit socially unacceptable behavior which may stem from impaired social cognition. A vast amount of evidence in the existing literature highlights the association between executive functions and theory of mind, focusing mainly on populations with autism spectrum disorders (see for example [13,14]; for a review on the relationship between executive functions and theory of mind in autism spectrum disorders see [15]; for a review about the parallel developmental course and association of the two constructs, see [16]. Taking into consideration the association between these two cognitive domains, it could be hypothesized that executive deficits in MMD patients could affect their social cognition. Moreover, there are clues of impaired social cognition in MMD patients based on clinical observation, which is also supported by a very limited number of studies. In our clinical practice, for example, we sometimes observe socially inappropriate verbal responses or even physical interaction (touching the medical equipment, being overfriendly or rude) from our patients. Such anecdotal reports are in accordance with scarce relevant findings in the literature. For example, Moore, Lee, & Macciocchi [17], in their MMD rehabilitation study, used a screening tool which included a subscale for social cognition, and identified patients with deficits in that domain. More recently, Araki and colleagues [18], utilized a more thorough neuropsychological approach by administering a theory of mind task to a group of MMD patients and concluded that a significant proportion of their group demonstrated relevant deficits. In accordance with the aforementioned notion of the linkage between theory of mind and executive functions, the above study by Araki et al. [18] provides direct evidence of impaired social cognition in MMD.

The current study aims to present a series of Greek patients with MMD, focusing on their neurocognitive profile including their social cognition capacity. To the best of our knowledge, this is the first study describing detailed neuropsychological data of MMD in the Greek population, and one of the very few reporting findings on social cognition of such patients.

## 2. Materials and Methods

### 2.1. Sample

The initial pool of patients consisted of 16 patients diagnosed with Μoyamoya Vasculopathy at the Stroke Unit of the Eginition University Hospital, Athens, Greece, from 2004–2020. The diagnosis was based on extra- and intra-cranial color-coded ultrasonography of the vascular network, magnetic resonance imaging (MRI) and angiography (MRA) and/or digital subtraction angiography (DSA), and in most cases CSF examination. Exclusion criteria implemented for this study were cranial trauma, alcohol or drug abuse, and visual or hearing impairment that could affect cognitive assessment. In total, eight patients (five women), 31–58 years old, were neuropsychologically assessed. Full neuropsychological data were obtained for six of them (see below, patients P1–P6). For the remaining two patients, a neuropsychological assessment was not completed due to time restrictions. This study was approved by the Hospital Ethics Committee and informed consent was attained from all patients (research protocol approval ID: ΩΣ3Ξ46Ψ8Ν2-00Φ, No 424, 21 July 2017).

### 2.2. Neuropsychological Assessment

**Figure 1:**
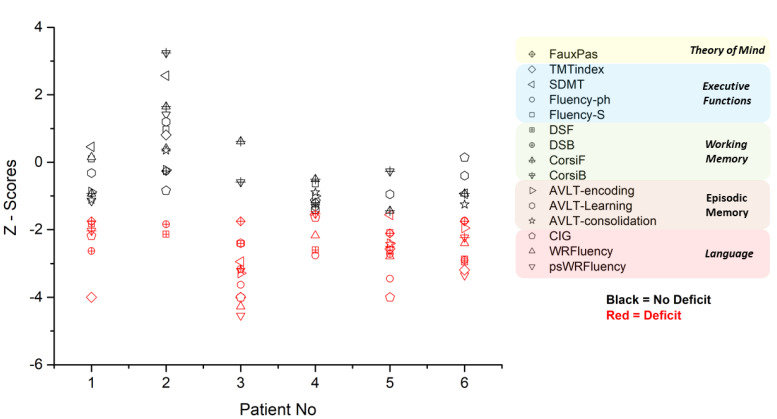
Cognitive performance (in *z—*scores) of the six patients with full neuropsychological data. Red indicates impaired performance (*z—*score at least 1.5 SD below the mean). Fluency*—*S z: *z—*scores for the semantic subscale of the Controlled Oral Word Fluency; Fluency*—*ph z: *z—*scores for the phonemic subscale of the Controlled Oral Word Fluency; WRFluency z: *z—*scores for the word reading fluency; psWRFluency z: *z—*scores for the pseudoword reading fluency; TMTindex z: *z—*scores for the Derived index of Trail Making Test; SDMT z: *z—*scores for the Symbol Digit Modality Test: AVLT*—*encoding z: *z—*scores for the encoding subscale of the Auditory Verbal Learning Test; AVLT*—*Learning z: *z—*scores for the learning subscale of the Auditory Verbal Learning Test; AVLT*—*consolidation z: *z—*scores for the consolidation subscale of the Auditory Verbal Learning Test; CIG z: *z—*scores for the Comprehension of Instructions in Greek; DSF z: *z—*scores for the Digit Span task Forward; DSB z: *z—*scores for the Digit Span task Backward; CorsiF z: *z—*scores for the Corsi Block*—*Tapping task Forward; CorsiB z: *z—*scores for the Corsi Block*—*Tapping task Backward; FauxPas z; *z—*scores for the Faux Pas Recognition Test. PPVT*—*R and BNT are not shown here, since for these tests only percentiles were available. However, impaired vs. non*—*impaired performance for these two tests is visualized in (Figure 2) (on the basis of a 5th percentile cut-off).

**Figure 2:**
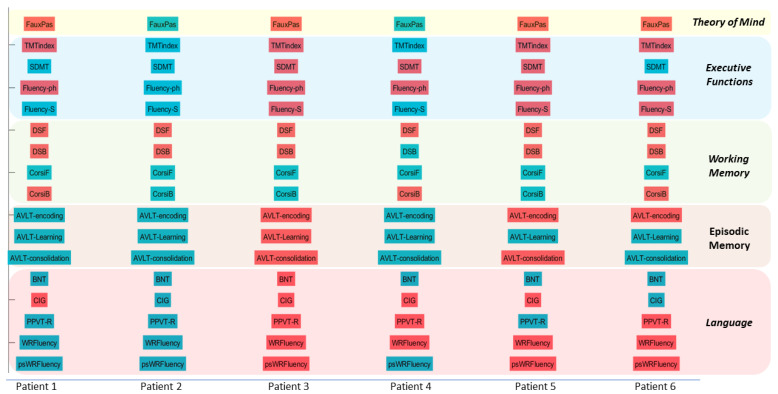
Neurocognitive profiles of the six patients with full neuropsychological data. Red indicates deficit. FauxPas: Faux Pas Recognition Test; ΤΜΤindex: Derived index for Trail Making Test; SDMT: Symbol Digit Modality Test; Fluency*—*ph: Controlled Oral Word Fluency, phonemic subscale; Fluency*—*S: Controlled Oral Word Fluency, semantic subscale; DSF: Digit Span task Forward; DSB: Digit Span task Backward; CorsiF: Corsi Block-Tapping task Forward; CorsiΒ: Corsi Block*—*Tapping task Backward; AVLT*—*encoding: Auditory Verbal Learning Test, encoding subscale; AVLT*—*Learning: Auditory Verbal Learning Test, learning subscale; AVLT*—*consolidation: Auditory Verbal Learning Test, consolidation subscale; BNT: Boston Naming Test; CIG: Comprehension of Instructions in Greek; PPVT*—*R: Peabody Picture Vocabulary Test–Revised; WRFluency: word reading fluency; psWRFluency: pseudoword reading fluency.

Neuropsychological testing was performed from 2018 to 2021 and included a comprehensive examination of the following cognitive domains: executive functions, memory, language, and social cognition. For executive functions, the Trail Making Test (TMT; [19]) was used to evaluate cognitive flexibility, the Symbol Digit Modality Test (SDMT; [20]) was used to evaluate processing speed, and the Controlled Oral Word Fluency (COWF; [21]) was used for assessment of semantic and phonemic fluency. Especially for TMT, a derived index was calculated to control for confounding factors such as motor deficits [22]. With regard to memory, episodic memory was assessed with the Greek version of the Auditory Verbal Learning Test (AVLT) [23] and for verbal and visual working memory with the Digit Span task (DS; [24]) and the Corsi Block-Tapping task [25,26]), respectively. Language examination included the Boston Naming Test (BNT; [27]) and the Peabody Picture Vocabulary Test-Revised (PPVT-R; [28]), both standardized in Greek [29,30], for assessing naming capacity and receptive vocabulary respectively, and Comprehension of Instructions in Greek (CIG; [31]) for assessing auditory comprehension of complex verbal material. In addition, we administered two standardized word and pseudoword reading fluency measures [32]. Social cognition was assessed with the Faux Pas Recognition Test (FP—[33,34]), adapted in Greek by Patrikelis and Angelakis [35,36]. Impaired performance was defined on the basis of either the fifth percentile or –1.5 standard deviation criteria, depending on the available normative data. For the Faux Pas Recognition Test, a group of 60 healthy participants served as a control group. Results of the neuropsychological assessment are described below and visualized in Figure 1 and Figure 2.

## 3. Results

### 3.1. Patient 1 (P2)

P1 (female) was diagnosed at the age of 40. She was 46 years old at the time of testing, with six years of education. Acute and chronic ischemic lesions were presented in the left hemisphere according to MRI findings. Semantic fluency was presented as normal, whereas phonemic fluency was deficient. Cognitive flexibility was also found to be impaired. Her processing speed was intact. Impaired performance was also noted in the auditory comprehension task, while reading and naming abilities, as well as receptive vocabulary, were preserved. In addition, the patient exhibited impaired performance in verbal and visual working memory tasks, while her episodic memory was normal. Finally, her performance in FP was lower than normal, thus indicating impaired social cognition.

### 3.2. Patient 2 (P2)

P2 (male) was diagnosed at the age of 54. At the time of the assessment, he was 58 years old with 13 years of education. The MRI findings did not reveal a stroke. The results of his cognitive examination indicated normal performance in all tests, except working memory tasks, in which he scored significantly lower than normal.

### 3.3. Patient 3 (P3)

P3 (female) was diagnosed at the age of 49. She was 50 years old at the time of testing, with 16 years of education. Her MRI finding indicated bilateral ischemic lesions. Her neuropsychological evaluation revealed impairment in all of the executive components tested, as well as in all indices of episodic memory. Naming, auditory comprehension, receptive vocabulary and reading fluency were also found to be impaired. With regard to working memory, deficits were found in the verbal but not in the visuospatial modality. Finally, the patient exhibited impaired social cognition, as indicated by her lower-than-expected performance in FP.

### 3.4. Patient 4 (P4)

P4 was diagnosed at the age of 41. She was 53 years old at the time of testing, with 12 years of education. Brain imaging revealed a left stroke. With regard to executive functions, deficits were found in phonemic fluency and processing speed, whereas semantic fluency and cognitive flexibility were found to be relatively preserved. Episodic memory was intact, while deficits were noted in verbal and visuospatial working memory. Lower than expected performance was demonstrated in language tasks related to receptive vocabulary, auditory comprehension, and word reading, while naming and pseudoword reading fluency were shown to be preserved. Social cognition was found to be unimpaired.

### 3.5. Patient 5 (P5)

P5 (male) was diagnosed at the age of 43. He was 46 years old at the time of testing with 12 years of education. An MRI revealed a left stroke. Patient’s scores on all executive tasks were found to be impaired. With regard to episodic memory, indices of encoding and consolidation were found to be significantly below normal, while learning capacity seemed to be relatively preserved. Working memory was impaired in the verbal modality, but not in the visuospatial modality. Normal performance was demonstrated in naming and receptive vocabulary tasks, whereas deficits were noted in auditory comprehension, as well as in both reading fluency subscales. His performance on FP was lower than expected, thus indicating deficits in social cognition.

### 3.6. Patient 6 (P6)

P6 (male) was 42 years old (diagnosed at the same age) at the time of testing, with 13 years of education. An MRI revealed a right stroke. His cognitive assessment revealed impairment in all executive functions, except processing speed. With regard to episodic memory, indices of learning and consolidation were found to be normal, but encoding was shown to be impaired. Deficits were also noted in both modalities of working memory. Receptive vocabulary was impaired, however normal performance was observed in auditory comprehension and in naming tasks. Reading fluency was impaired for both words and pseudowords. Deficits were also noted in social cognition.

### 3.7. Patient 7 (P7)

P7 (female) was 50 years old (diagnosed at the same age) at time of testing, with 12 years of education. An MRI revealed an acute bilateral ischemic lesion. Besides phonemic fluency, which was found to be impaired, her performance was within normal limits in the rest of the executive tests (processing speed data are not available for this patient). Language assessment did not reveal any deficit except for word reading fluency. Episodic memory, working memory, and social cognition were not assessed.

### 3.8. Patient 8 (P8)

P7 (female) was diagnosed with Moyamoya syndrome at the age of 30. She was 31 years old at the time of testing, with 16 years of education. An MRI showed a left-lateralized acute ischemic lesion. With regard to executive functions, normal performance was shown in tasks of semantic fluency and cognitive flexibility, while phonemic fluency was found to be impaired. Her processing speed was not assessed. With regard to language, receptive vocabulary, auditory comprehension, and word and pseudoword reading fluency were found to be impaired, while naming ability was relatively preserved. Episodic memory, working memory, and social cognition were not assessed.

## 4. Discussion

The aim of the current study was the presentation of a case-series of Moyamoya Greek patients, focusing on the neuropsychological manifestations of the disease. The sample may be small, thus this is not presented as a group study, but rather as a series of patients. However, it should be noted that this particular neurological condition is rather rare among Caucasians, and, to the best of our knowledge, there are no published reports on the cognitive impairment of Greek patients. We therefore hope that this study will constitute the initiation of longitudinal collection of data focused on—but not necessarily limited to the burden of Moyamoya Disease on cognition in the Greek population.

Even though cognitive impairment has been well documented in children with MMD, data on adults, and especially Caucasians, are not sufficient to provide a detailed overview of the cognitive status of such patients, and only general conclusions have been drawn, such as, for example, the fact that there are differences between adult and pediatric populations (for a review, see [37]). Consequently, there is still no clear consensus on the cognitive profile of adult patients. Therefore, understanding the full scope of the impact of MMD on different aspects of cognition would require a detailed neuropsychological assessment. In the current study, such an assessment revealed that MMD may cause cognitive impairment in specific cognitive domains in adults.

With regard to the cognitive profile of our patients, most prominent deficits were identified in executive functions, social cognition and verbal working memory, whereas other aspects of cognition, such as episodic memory, seemed to remain unaffected by the neurological manifestations of the disease (see Figure 1 and Figure 2).This cognitive profile is generally in accordance with findings of previously published case reports [9] and group studies [11,38], which highlight the prominence of executive dysfunction in MMD. It should be noted, however, that even though most of our patients exhibited executive deficits, they exhibited within normal range performance in certain tasks, like SDMT (e.g., P1 and P6). Regarding specific neuropsychological scores evaluated in MMD, there are conflicting reports in the relevant literature. For example, all of our patients except P2 showed impaired performance on the phonemic fluency task, which is in accordance with some previously published findings [12] but contradicts other studies [39]. Such discrepancies could be attributed to the differences between these studies with regard to the neuropsychological tests used and their clinical sample. While Karzmark and colleagues [12] administered a fluency test with three subscales corresponding to the letters F, A, and S, Calviere and colleagues [39] administered a different version of a fluency task that involved only one letter. In addition, while the first study [12] included a significant proportion of patients with naming deficits, in the latter research report [39], only one out of ten patients exhibited such difficulties. Naming deficits could reflect impaired lexical access, which in turn could negatively affect word retrieval, resulting in lower scores in fluency tasks.

**Figure 3:**
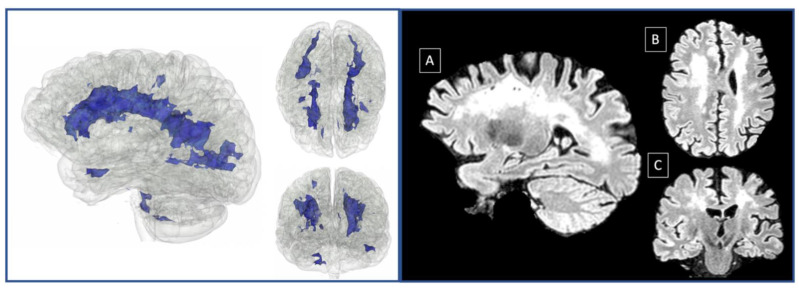
White matter lesions 3D model in native space (left). Saggital (**A**), axial (**B**) and coronal (**C**) view of 3D Flair in native space (right). The three-dimensional (3D) brain model was built from a patient’s (P6) 3D Flair (Repetition time (TR): 4800 ms, Echo Time (|TE): 284 ms, slice thickness 1.48 mm, flip angle: 90, sagittal orientation) in native space, using the Model Maker Module, in 3D slicer version 4.11.2 [56]. White matter lesions were automatically detected with 3D Flair as input, using Lesion Spotlight extension [57,58] run on 3D slicer.

Only P3 exhibited an impaired performance in episodic memory, which could be at least partly attributed to her generalized cognitive decline. As for the language domain, there were patients who showed deficits in several linguistic aspects; however, we could only speculate about their origin. For example, for P1, P3, P4, and P5, impaired scores could be attributed to the left-lateralized ischemic lesions, but this was not the case for P6. An alternative explanation for language deficits could be based on the diffuse white matter lesions found in MMD patients (see Figure 3). Finally, verbal working memory was found to be systematically affected in our group.

Overall, our study confirms previous reports highlighting executive impairment in MMD, and adds additional information about the cognitive profiles of such patients by revealing deficits in working memory and social cognition. Here, it should be noted that there are sparse studies assessing social cognition of MMD patients [17,18]. To the best of our knowledge, this is the first study reporting impaired performance in social cognition, and particularly theory of mind, in a case series of Caucasians with MMD. A lingering question concerns the co-occurrence of deficits in executive functions, working memory, and social cognition in MMD. A possible explanation of the observed co-morbidity of impaired social cognition and dysexecutive phenomenology could be based on the fact that executive functions are presumably associated with theory of mind in terms of anatomy and function [34]. A more through hypothesis could be formulated on the basis of the anatomical substrate of working memory, social cognition, and executive functions, in conjunction with the lesional pattern of MMD. Brain imaging studies point to a widely distributed network, comprising of posterior, anterior, as well as underlying white matter, which supports executive functions ([40,41]; for a review, see [42]). On the other hand, meta-analyses highlight the importance of a fronto-parietal network as the neurobiological substrate of WM [43,44]. This notion is further supported by studies investigating individual components of working memory, and demonstrates that these are associated with the posterior parietal cortex, as well as the dorsolateral and ventrolateral prefrontal cortex [45,46,47]. With regard to the neural correlates of social cognition, the reported evidence may be yet inconclusive, but the emerging trend is in favor of a distributed network supporting this particular psychological construct, since brain imaging studies reveal that performance on relevant tasks (e.g.,theory of mind tests) elicit hemodynamic responses in the medial prefrontal cortex, the orbitofrontal cortex, the temporal pole, the temporoparietal junction, and the superior temporal sulcus [48,49,50,51,52,53]. In sum, all of the aforementioned cognitive domains cannot be linked to a single brain area but are rather associated with a broader network of cortical and subcortical brain regions. MMD, on the other hand, is a neurological disorder that results in diffuse lesions mostly affecting white matter tracts (see Figure 3). In this sense, following Campbell’s hodological approach, according to which corticocortical connectivity is crucial for higher cognitive functions [54], we could adopt the hodotopic framework proposed by Catani and Ffytche [55]. Within this framework, the neurocognitive pattern of MMD could be interpreted as a result of white matter lesions (in synergy with cortical lesions due to circumstantial strokes), that consequently affect the above-described networks supporting the three cognitive domains. This view is in line with evidence showing that cognitive dysfunction can be seen also in MMD patients without stroke, i.e., without cortical damage [12,38].

At this point, we should note the limitations of this study. The sample is small, given the rarity of the disease, especially in Caucasians. Due to the small sample size and the lack of consistent neuroimaging data from a single MRI scanner, we were not able to perform statistical analyses in order to clarify crucial aspects, such as the possible effect of the strokes that some of our patients suffered, taking into consideration lesion-related variables, and eventually reach conclusions about the unique contribution of focal lesions and the disease per se. Moreover, due to the small sample size, we cannot generalize our findings to the MMD clinical population, although the general neurocognitive pattern presented here is in accordance with previous studies.

Overall, this is one of the few studies reporting detailed neuropsychological data on Caucasian MMD patients, and, to the best of our knowledge, the first study with Greek patients. Our preliminary data show frequently demonstrated impairment in three cognitive domains—namely working memory, executive functions, and social cognition—which could be interpreted on the basis of diffuse lesions affecting widely distributed networks supporting these cognitive functions. Finally, the present case series is one of the very few reports highlighting the importance of social cognition in MMD, and we argue that this specific issue should be further investigated in larger clinical samples. Acknowledging the rareness of the disease, future studies may also include neuroimaging data to further investigate relations between the aforementioned cognitive deficits and the quantitative indices of lesions.

## Data Availability

The data presented in this study are available on request from the corresponding author. The data are not publicly available due to ethical reasons related to patient confidentiality.
